# Tumor Suppressive Function of NQO1 in Cutaneous Squamous Cell Carcinoma (SCC) Cells

**DOI:** 10.1155/2019/2076579

**Published:** 2019-11-22

**Authors:** Qing-Ling Zhang, Xue Mei Li, De-De Lian, Ming Ji Zhu, Su-Hyuk Yim, Jeung-Hoon Lee, Ri-Hua Jiang, Chang-Deok Kim

**Affiliations:** ^1^Department of Dermatology, China-Japan Union Hospital of Jilin University, Changchun, Jilin, China; ^2^Department of Dermatology, School of Medicine, Chungnam National University, Daejeon, Republic of Korea; ^3^Department of Medical Science, School of Medicine, Chungnam National University, Daejeon, Republic of Korea; ^4^Department of Intensive Care Unit, China-Japan Union Hospital of Jilin University, Changchun, Jilin, China; ^5^Skin Med Company, Daejeon, Republic of Korea

## Abstract

Cutaneous squamous cell carcinoma (SCC) is a common cancer that significantly decreases the quality of life. It is known that external stimulus such as ultraviolet (UV) radiation induces cutaneous SCC via provoking oxidative stress. NAD(P)H dehydrogenase 1 (NQO1) is a ubiquitous flavoenzyme that functions as a guardian against oxidative stress. However, the effect of NQO1 on cutaneous SCC is not clearly elucidated. In this study, we investigated the effect of NQO1 on cutaneous SCC cells using the recombinant adenoviruses that can upregulate and/or downregulate NQO1 expression. Overexpression of NQO1 resulted in significant decrease of cell proliferation and colony forming activity of SCC lines (SCC12 and SCC13 cells). By contrast, knockdown of NQO1 increased the cell proliferation and colony forming activity. Accordingly, the levels of proliferation-related regulators, such as Cyclin D1, Cyclin E, PCNA, SOX2, and p63, were decreased by the overexpression of NQO1, while those were increased by knockdown of NQO1. In addition, NQO1 affected the invasion and migration of SCC cells in a very similar way, with the regulation of epithelial-mesenchymal transition- (EMT-) related molecules, including E-cadherin, N-cadherin, Vimentin, Snail, and Slug. Finally, the overexpression of NQO1 decreased the level of phosphorylated AKT, JNK, and p38 MAPK, while the knockdown of NQO1 increased the level of phosphorylated signaling molecules. Based on these data, NQO1 has tumor suppressive function in cutaneous SCC cells.

## 1. Introduction

Cutaneous squamous cell carcinoma (SCC) is a common cancer, which is originated from the differentiated keratinocytes in upper layers of epidermis. It is the second most frequent type among the nonmelanoma skin cancers, influencing the quality of life considerably [1, 2]. Many factors are known to affect the development of cutaneous SCC. The most important environmental risk factor is ultraviolet (UV) radiation that manifests its possible detrimental effect via the production of reactive oxygen species (ROS) [3, 4]. In addition, many intracellular regulators such as epidermal growth factor receptor (EGFR), tumor protein p53 (TP53), and Wnt/*β*-catenin signaling pathways are implicated in the pathogenesis of cutaneous SCC [5–9].

NAD(P)H dehydrogenase 1 (NQO1) is a ubiquitous flavoenzyme that catalyzes the two-electron reduction of quinones to hydroquinones using NAD(P)H as an electron donor [10, 11]. NQO1 functions as a guardian against oxidative stress in various ways, such as detoxifying the highly reactive quinones, maintaining lipid-soluble antioxidants in reduced forms, and stabilizing the tumor suppressor p53 [12]. It has been reported that the deletion of NQO1 gene makes mice more susceptible to oxidative stress and frequently develop skin tumor upon carcinogen exposure [13]. By contrast, it has been also reported that the knockdown of NQO1 diminishes the proliferation of glioblastoma cells while the overexpression increases the cell proliferation [14]. Based on these opposite data, NQO1 is thought to possess both the pro- and/or antitumorigenic roles depending on different conditions and cell types.

As the development and progression of cutaneous SCC are closely related to the UV-induced oxidative stress, we speculate that NQO1 may have roles in SCC. In this study, we demonstrate the tumor suppressive function of NQO1 in cutaneous SCC cells.

## 2. Materials and Methods

### 2.1. Immunohistochemistry

Skin tissues were fixed in 10% (v/v) formaldehyde and embedded in paraffin. The paraffin-embedded sections of skin specimens were dewaxed, rehydrated, and washed three times with phosphate-buffered saline (PBS). Sections were then incubated with proteinase K (Dako, Carpinteria, CA, USA) for 5 min at 37°C, treated with H_2_O_2_ for 10 min at room temperature, and blocked in 0.1% Tween-20 (v/v) and 1% bovine serum albumin (BSA) in PBS (w/v) for 30 min, and this was followed by reaction with anti-NQO1 antibody (Santa Cruz Biotechnology, Santa Cruz, CA) for 1 h. Sections were incubated sequentially with peroxidase-conjugated secondary antibody (Dako) and visualized with a ChemMate EnVision detection kit (cat# K5007) (Dako).

### 2.2. Cell Culture

SCC12 and SCC13 cells are the human squamous cell carcinoma line, established from SCCs of the facial epidermis [15]. Both the cells were maintained in Dulbecco's modified Eagle's medium (DMEM) supplemented with 5% fetal bovine serum (FBS) (v/v) (Life Technologies Corporation, Grand Island, NY). Simian virus 40 large T antigen-transformed human epidermal keratinocytes (SV-HEK) were maintained in keratinocyte-serum-free medium supplemented with bovine pituitary extract and recombinant human epidermal growth factor (Life Technologies Corporation) [16]. Human dermal fibroblasts were primary cultured and maintained in DMEM supplemented with 10% FBS.

### 2.3. Production of Recombinant Adenovirus

The NQO1 cDNA was obtained by reverse transcription-polymerase chain reaction (RT-PCR). Briefly, total RNA was isolated using the Easy-blue RNA extraction kit (Intron, Daejeon, Korea). Two micrograms of total RNA was reverse-transcribed with Moloney murine leukemia virus reverse transcriptase (Elpis Biotech, Daejeon, Korea). An aliquot of the RT mixture was subjected to PCR with the primer set for NQO1 (5′-ATGGTCGGCAGAAGAGCACTGA and 5′-CAGATCAAAGCTAGAAAATGA). The amplified full-length NQO1 cDNA was subcloned into the pENT/CMV vector, and replication-incompetent adenoviruses were created [17].

For knockdown experiments, we prepared recombinant adenoviruses expressing a microRNA targeting NQO1. The target sequences for NQO1 were designed using BLOCK-iT™ RNAi Designer (Thermo Scientific, Rockford, IL). The double-stranded DNA oligonucleotides were synthesized and cloned into the parental vector pcDNA6.2-GW/EmGFP-miR (Thermo Scientific). The expression cassette for microRNA was inserted into the pENT/CMV vector, and then the adenovirus was prepared using the method described above. The microRNA sequences are as follows: top strand 5′- TGCTGTTCAGTTTACCTGTGATGTCCGTTTTGGCCACTGACTGACGGACATCAGGTAAACTGAA, bottom strand 5′-CCTGTTCAGTTTACCTGATGTCCGTCAGTCAGTGGCCAAAACGGACATCACAGGTAAACTGAAC.

### 2.4. Western Blot

Cells were lysed in PRO-PREP solution (Intron). Total protein concentrations were measured using a BCA protein assay kit (Thermo Scientific, cat# 23225). Samples were run onto SDS-polyacrylamide gels and transferred onto nitrocellulose membranes (Pall Corporation, Port Washington, NY). After blocking with 5% skim milk (w/v), the membranes were incubated with primary antibodies. Blots were then incubated with peroxidase-conjugated secondary antibodies and visualized using enhanced chemiluminescence (Intron). The following primary antibodies were used: PCNA (cat# SC-7907), NQO1 (cat# SC-32793), p-ERK (cat# SC-7383), and *β*-Actin (cat# SC-47778) (Santa Cruz Biotechnology, Santa Cruz, CA); Cyclin D1 (cat# 2978S), Cyclin E1 (cat# 4129S), SOX2 (cat# 2748S), E-cadherin (cat# 3195S), N-cadherin (cat# 4061S), Vimentin (cat# 5741S), Snail (cat# 3895S), p-JNK (cat# 4668S), p-AKT (cat# 4060), and p-p38 (cat# 9216S) (Cell Signaling Technology, Danvers, MA); and p63 (cat# ab53039) and Slug (cat# ab27568) (Abcam, Cambridge, UK).

### 2.5. Cell Growth Assay and Colony Forming Assay

For cell growth assay, SCC12 and SCC13 cells were transduced with 10 multiplicity of infection (MOI) of adenovirus for overnight. Cells were replenished with fresh medium and incubated for a further 2 d. Cells were then trypsinized and 1 × 10^4^ cells were reseeded on each 100 mm culture dishes. At the indicated time points, cells were trypsinized and cell numbers were counted using hemocytometer.

For colony forming assay, 1 × 10^3^ cells were reseeded on each 100 mm culture dishes. Cells were grown for 2–3 weeks and stained with crystal violet (Sigma, St. Louis, MO).

### 2.6. Invasion Assay

Invasion assay was performed using the Chemicon Cell Invasion Assay Kit (cat# ECM550) (Merck KGaA, Darmstadt, Germany). Briefly, after transduction with adenovirus, cell suspension was prepared in serum-free medium and added to upper chamber. Lower chamber received 5% FBS containing medium. After incubation for 48–72 h, noninvading cells and ECMatrix were removed using cotton-tipped swabs. Invading cells were visualized by staining solution.

### 2.7. Scratch Wound Assay

After adenoviral transduction, cells were cultured for 2 d in growth medium. Cells were then replenished with serum-free medium, incubated for 6–8 h, and treated with mitomycin C (10 *μ*g/ml) for 2 h. After changing the medium supplemented with 5% FBS, thin wound was introduced by scratching with a pipette tip. Cell migration was evaluated using ImageJ program. Wound closure was determined by calculating the proportion of wound size at 12 h to initial wound.

### 2.8. Detection of Cellular ROS

Cells were transduced with adenovirus and cultured for 2 d. Cells were then washed 3 times with Hank's balanced salt solution (HBSS) and incubated with ROS Deep Red dye (cat# ab186029) (Abcam) for 1 h. Cells were then observed under the fluorescent microscopy.

### 2.9. Statistical Analysis

Data were evaluated statistically by one-way ANOVA or Student's *t*-test using SPSS software v 22.0 (IBM, Seoul, Korea). Statistical significance was set at *p* < 0.05.

## 3. Results

**Figure 1:**
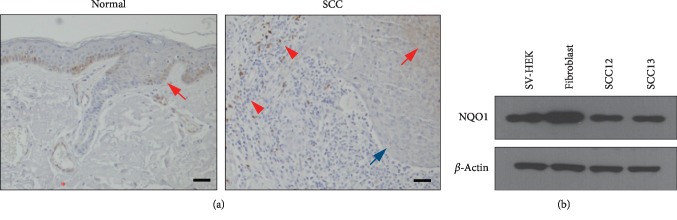
Expression of NQO1 in cutaneous SCC. (a) Normal and SCC lesional areas were obtained from the same patients, and skin specimens were immunohistochemically stained using anti-NQO1 antibody. Scale bar: 100 *μ*m. (b) Expressions of NQO1 in cultured skin cells were verified by Western blot. SV-HEK: simian virus 40 large T antigen- (SV40T) transformed human epidermal keratinocytes; SCC12 and SCC13: cutaneous squamous cell carcinoma cell lines.

We examined the expression level of NQO1 by immunohistochemistry in the normal and SCC lesional area obtained from the same patient. NQO1 immunoreactivity was observed in the epidermis (red arrows) and vessels (red asterisks) of normal region of SCC patient. By contrast, NQO1 was barely detected (blue arrows) or partially detected (red arrows) in the lesional area of SCC. NQO1 immunoreactivity was also observed in immune cells surrounding SCC lesion (red arrowheads) (Figure 1(a)). In cultured cutaneous SCC cells (SCC12 and SCC13) and skin-comprising cells, the level of NQO1 protein was slightly lower in SCC cells compared to keratinocytes and fibroblasts (Figure 1(b)).

**Figure 2:**
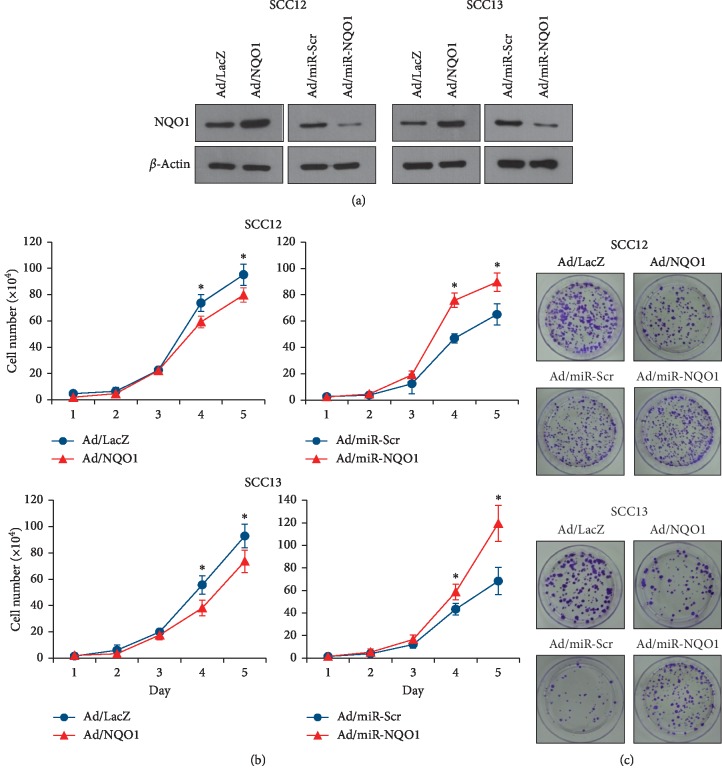
Effect of NQO1 on cell proliferation and colony forming activity. (a) SCC12 and SCC13 cells were transduced with the adenoviruses expressing NQO1 (Ad/NQO1) and/or a microRNA targeting NQO1 (Ad/miR-NQO1). The adenoviruses expressing LacZ (Ad/LacZ) and/or miR-scrambled (Ad/miR-Scr) were used for negative controls. Western blots show effective overexpression and/or knockdown of NQO1 in both the cell lines. (b) Effect of NQO1 on cell proliferation. Overexpression of NQO1 decreased the cell proliferation, whereas knockdown of NQO1 increased the cell proliferation. The mean values ± SD are averages of triplicate measurements. ^*∗*^*p* < 0.05. (c) Colony forming assay. Overexpression of NQO1 decreased the colony forming activity, while knockdown of NQO1 increased the colony forming activity.

To investigate the potential role of NQO1 in SCC cells, we created the recombinant adenovirus expressing NQO1 (Ad/NQO1). We also created the recombinant adenovirus expressing a microRNA targeting NQO1 (Ad/miR-NQO1) to downregulate its expression. After transduction with the Ad/NQO1, NQO1 was expressed at high level in SCC cells compared with the control adenovirus (Ad/LacZ)-treated group. By contrast, the level of the NQO1 protein was markedly decreased after the transduction of Ad/miR-NQO1 compared to cells transduced with the control adenovirus (Ad/miR-Scr) (Figure 2(a)). We then investigated whether NQO1 affected the cell proliferation. When NQO1 expression was increased, the cell proliferation was decreased in both the SCC12 and SCC13 cells. In contrast to NQO1 overexpression, downregulation of NQO1 increased the cell proliferation (Figure 2(b)). Next, we determined the colony forming activity, which manifested the tumorigenic potential in vitro condition [18]. Similar to the results obtained from cell proliferation assay, the overexpression of NQO1 decreased the colony forming activity while knockdown of NQO1 increased the colony forming activity in both the SCC12 and SCC13 cells (Figure 2(c)).

**Figure 3:**
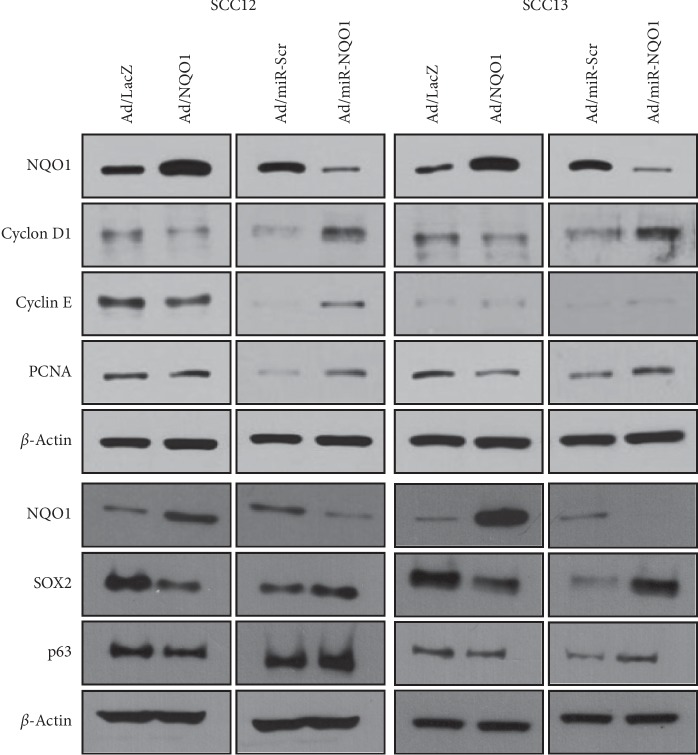
Effect of NQO1 on the level of cell proliferation-related molecules. After adenoviral transduction, cells were cultured for 2 d. Overexpression of NQO1 decreased the level of Cyclin D1, Cyclin E, PCNA, SOX2, and p63 protein, whereas knockdown of NQO1 increased the level of those proteins.

We assessed whether NQO1 affected the cell proliferation-related regulators. The overexpression of NQO1 significantly decreased the level of several regulators, such as Cyclin D1, Cyclin E, PCNA, SOX2, and p63. By contrast, miR-mediated downregulation of NQO1 increased the level of cell proliferation-related regulators (Figure 3).

**Figure 4:**
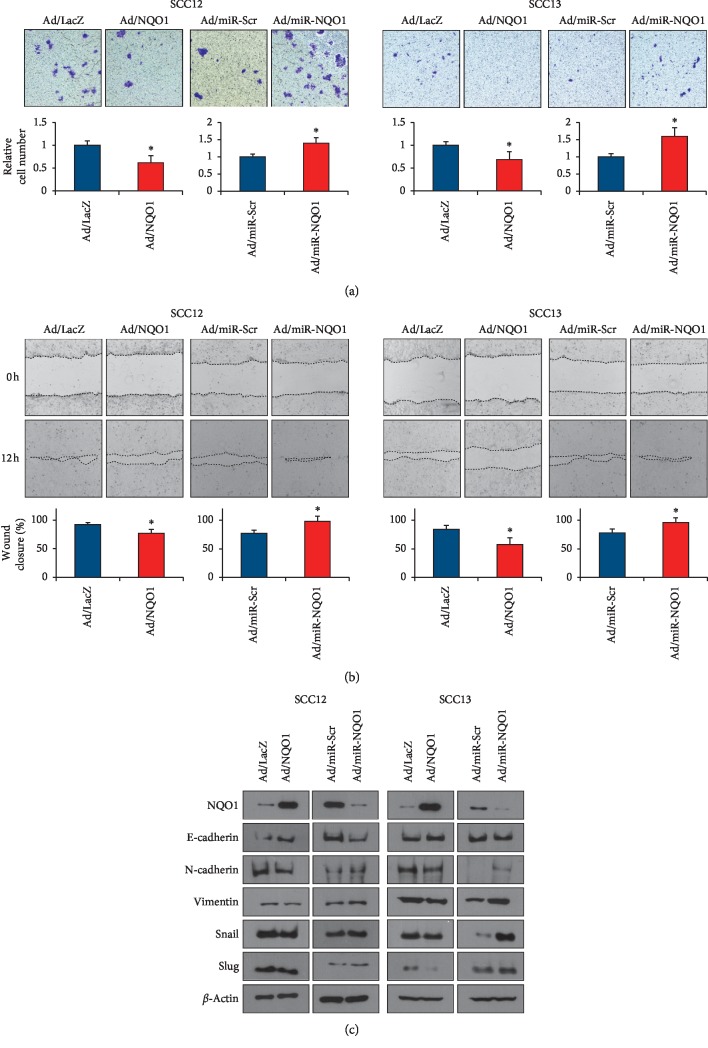
Effect of NQO1 on invasion and migration. (a) After adenoviral transduction, invasion assay was performed. Overexpression of NQO1 decreased the invasion, while knockdown of NQO1 increased invasion of SCC cells. The mean values ± SD are averages of triplicate measurements. ^*∗*^*p* < 0.05. (b) After adenoviral transduction, scratching wound was created using a pipette tip. Wound closure was determined by calculating the proportion of wound size at 12 h to initial wound. The mean values ± SD are averages of triplicate measurements. ^*∗*^*p* < 0.05. (c) Effect of NQO1 on the molecules related to epithelial-mesenchymal transition (EMT). Overexpression of NQO1 decreased the level of EMT markers, whereas knockdown of NQO1 increased the level of EMT markers.

As the invasive growth and migration are the important manifestations of tumor progression, we next investigated whether NQO1 affected those characteristics of SCC cells. The overexpression of NQO1 significantly reduced the invasion of SCC cells, while the knockdown of NQO1 increased the invasion of SCC cells (Figure 4(a)). Similarly, cell migration was also decreased by NQO1 overexpression but increased by NQO1 downregulation (Figure 4(b)). We then checked the effect of NQO1 on epithelial-mesenchymal transition- (EMT-) related molecules. It has been recognized that the loss of E-cadherin is a fundamental event in EMT, whereas the level of several molecules such as N-cadherin, Vimentin, Snail, and Slug are increased in this process [19]. The overexpression of NQO1 increased the level of E-cadherin, while it slightly decreased the level of N-cadherin, Vimentin, Snail, and Slug. By contrast, the knockdown of NQO1 slightly decreased the level of E-cadherin, while it increased the level of other molecules (Figure 4(c)).

**Figure 5:**
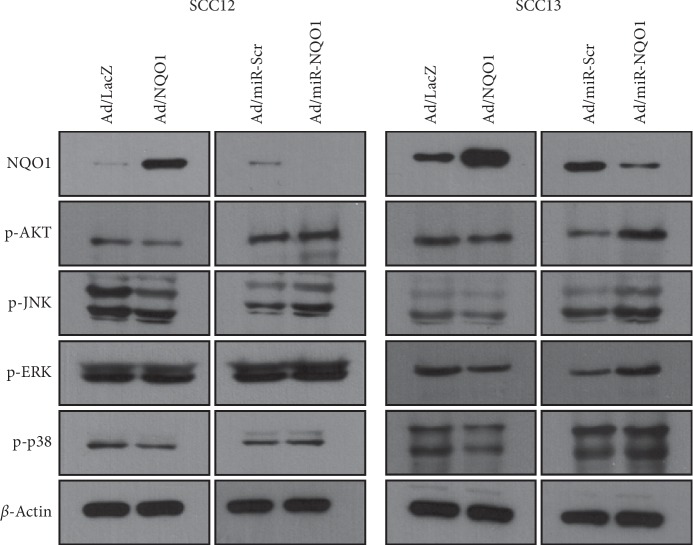
Effect of NQO1 on the intracellular signaling molecules. After adenoviral transduction, cells were cultured for 2 d. Overexpression of NQO1 decreased the level of phosphorylated AKT, JNK, and p38 MAPK, whereas knockdown of NQO1 increased the level of those signaling molecules.

Several signaling pathways are implicated in EMT. For example, AKT and mitogen-activated protein kinase (MAPK) pathways are involved in EMT of esophageal SCC [20]. Thus, we examined whether NQO1 affected intracellular signaling pathways related to EMT. The overexpression of NQO1 slightly decreased the level of phosphorylated AKT, JNK, and p38 MAPK, whereas the knockdown of NQO1 increased the level of phosphorylated AKT, JNK, and p38 MAPK. The effect of NQO1 on ERK was not obvious (Figure 5).

**Figure 6:**
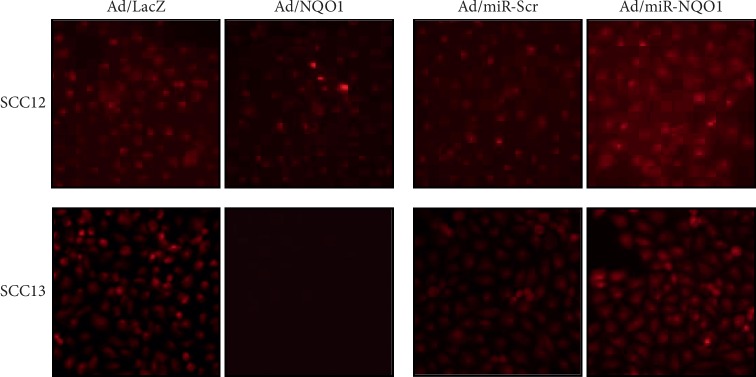
Effect of NQO1 on cellular ROS level. After adenoviral transduction, cells were cultured for 2 d and cellular ROS level was measured using Deep Red dye. Overexpression of NQO1 decreased the ROS level, whereas knockdown of NQO1 increased it.

To investigate the putative mechanism underlying NQO1-induced tumor suppressive effect, we determined the cellular ROS level after the overexpression or knockdown of NQO1. When NQO1 was overexpressed, cellular ROS level was markedly decreased. By contrast, the knockdown of NQO1 resulted in significant increase of ROS level (Figure 6). These results suggest that NOQ1 regulates cellular ROS level, thereby affecting the cancerous phenotype in cutaneous SCC cells.

**Figure 7:**
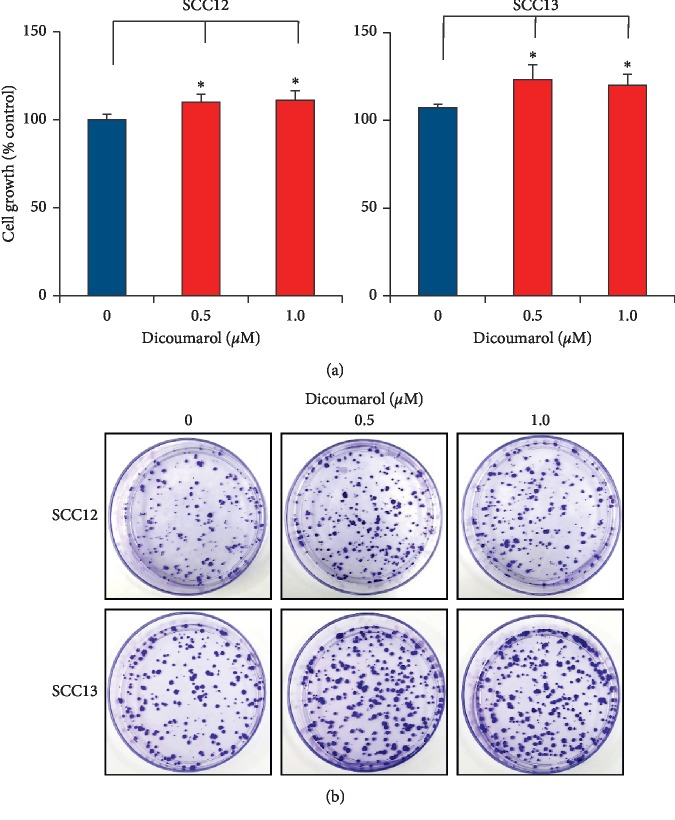
Effect of dicoumarol, NQO1 inhibitor, on cell growth and colony forming activity. (a) Cells were treated with dicoumarol at the indicated concentration for 24 h. Cell growth was measured by MTT assay. Dicoumarol significantly increased the cell growth. Data are expressed as percentage of control. The mean values ± SD are averages of triplicate measurements. ^*∗*^*p* < 0.05. (b) Dicoumarol increased the colony forming activity of SCC cells.

Finally, dicoumarol, NQO1 inhibitor, significantly increased the cell growth at the concentration of 0.5 and 1.0 *μ*M. Consistent with these data, dicoumarol increased the colony forming activity (Figure 7). These results support the idea that tumor suppressive effect is related to antioxidant role of NQO1.

## 4. Discussion

In this study, we demonstrated the tumor suppressive function of NQO1 in cutaneous SCC cells. When NQO1 was overexpressed, the cell proliferation and colony forming activity were decreased. By contrast, the knockdown of NQO1 led to the increase of cell proliferation and colony forming activity. Similarly, the invasion and migration of SCC cells were decreased by NQO1 overexpression, whereas they increased by NQO1 knockdown. In addition, the levels of proliferation- and EMT-related molecules are affected in a similar way. These results support the notion that NQO1 has tumor suppressive function in SCC cells.

NQO1 is a flavoenzyme that exerts its role in the cellular defense mechanism against oxidative stress. As the physiological function of NQO1 is to detoxify the potential mutagenic compounds, it can be speculated that the decrease of NQO1 may predispose the cells to more susceptible condition to cancer development. This idea is supported by the fact that NQO1 knockout mice develop skin tumor at high frequency when carcinogens are applied [13]. In other example, individuals with a lack of NQO1 due to a genetic polymorphism show an increased susceptibility to certain cancers [21]. Additionally, the overexpression of NQO1 inhibits hepatocellular carcinoma cell proliferation through AMPK/PGC-1*α* pathway [22]. However, there are many conflicting reports regarding the role of NQO1 in cancer development. For instance, high-level expression of NQO1 appears to be associated with breast cancer progression [23]. In other example, NQO1 could potentiate non-small-cell lung cancer (NSCLC) cell proliferation by enhancing cellular glycometabolism [24]. Therefore, it can be assumed that NQO1 has the dual roles with pro- and/or antitumorigenic potential depending on different conditions and cell types. In this study, we showed that NQO1 suppressed tumor characteristics in cutaneous SCC cells, suggesting that NQO1 functions as an antitumorigenic regulator in SCC development and progression.

The potential role of AKT in SCC has been demonstrated several times in other systems. For example, codeletion of p53 and *α*v integrin genes in mouse stratified epithelia induces SCCs via the activation of AKT [25]. In other example, UV radiation promotes AKT activation via the induction of stress-inducible protein Sestrin2 (SESN2), contributing to the survival of SCC cells [26]. Finally, tumor induction by 7,12-dimethylbenz(a)anthracene in conditional TGF-*β* receptor 1 (Tgfbr1) knockout mice is mediated through the activation of AKT pathway [27]. Similarly, MAPK signaling pathways are well implicated in cell migration and EMT. For instance, UV radiation induces EMT-related molecules including Snail and Slug via the activation of ERK and p38 MAPK in keratinocytes [28]. Other evidence shows that TGF-*β*1-induced fascin1 facilitates the migration and invasion of kidney carcinoma cells through ERK and JNK signaling pathways [29]. In this study, the overexpression of NQO1 decreased the level of phosphorylated AKT, JNK, and p38 MAPK, while knockdown increased their level. Therefore, it can be suggested that the regulation of AKT and MAPK signaling pathways by NQO1 is one putative mechanism underlying tumor suppressive function of NQO1. Elucidation of precise link between NQO1 and intracellular signaling pathways will be an interesting further study.

In summary, we demonstrate that NQO1 has tumor suppressive function in cutaneous SCC cells. Our results provide new insights on which we base further investigations of the molecular events underlying SCC development.

## Data Availability

The datasets generated and/or analyzed during the current study are available from the corresponding author upon reasonable request.
